# SAHELI:  Study and Action on Hysterectomy: Evidence on women’s health through the life course in India.  Protocol for a mixed-methods study

**DOI:** 10.12688/wellcomeopenres.23084.2

**Published:** 2025-03-05

**Authors:** Sapna Desai, Dipti Govil, Devaki Nambiar, Hemali Heidi Sinha, Archana Roy, Kranti Vora, Josyula K Lakshmi, Archana Kumari, Gita D Mishra, Neerja Bhatla

**Affiliations:** 1 Population Council Institute, New Delhi, Delhi, India; 2 International Institute for Population Sciences, Mumbai, Maharashtra, India; 3 The George Institute for Global Health India, New Delhi, Delhi, India; 4 All India Institute of Medical Sciences Patna, Patna, Bihar, India; 5 Independent Consultant, Gandhinagar, Gujarat, India; 6 All India Institute of Medical Sciences Delhi, New Delhi, Delhi, India; 7 University of Queensland School of Public Health, Herston, Australia

**Keywords:** hysterectomy, women's health, life course, health systems, India, gynaecology, India

## Abstract

Hysterectomy, removal of the uterus, is a commonly performed surgery for gynaecological morbidities. Emerging evidence indicates that hysterectomy performed before age 45 (early hysterectomy), is associated with considerable risks to women’s health. While most evidence on hysterectomy is from high-income settings, national surveys from India report high prevalence of early hysterectomy in specific regions, as well as higher prevalence amongst women in rural areas and with less education. The median age at hysterectomy in India is close to ten years before the onset of natural menopause. India has recently introduced national guidelines to address early hysterectomy, but large evidence gaps on the causes and consequences remain – which in turn limits the potential effectiveness of interventions at the clinical, health system and community level.

**Methods:**

SAHELI is a Team Science study that will examine: (i) individual, social and health system determinants of early hysterectomy; (ii) women’s treatment pathways to hysterectomy and for gynaecological morbidity in general; and (iii) the consequences of undergoing hysterectomy on women’s physical, mental, economic and social well-being across the life course. This mixed-methods study includes population surveys amongst women in ages 25–49 in three high-prevalence states; qualitative health systems research to trace treatment journeys with women, health care providers and other stakeholders; evidence syntheses; and knowledge translation activities to ensure findings inform co-produced strategies and interventions. The study is grounded in a feminist epidemiology approach, aiming to examine individual and structural causes of vulnerability and prioritising the views of women, particularly in knowledge translation.

**Conclusions:**

SAHELI, implemented by an all-women, multi-disciplinary team, is the first study in India to examine the causes and consequences of hysterectomy in a life course approach. We aim to influence interventions, policy and future research on women’s health, particularly access to quality gynaecological care and comprehensive health services through the life course.

## Plain language summary

Hysterectomy, the surgical removal of the uterus, is one of the most common surgeries performed on women. While it can be life-saving for specific conditions, hysterectomy conducted much before natural menopause (early hysterectomy) can also have negative consequences on women’s health, such as increased risk of cardiovascular disease or osteoporosis. Most research on hysterectomy is from high-income countries. However, recent national studies in India indicate high rates of hysterectomy in some regions, amongst women in their mid-thirties - at least a decade before natural menopause. It is also more common amongst disadvantaged women in rural areas and with less education.

We have initiated a new study, SAHELI, to investigate the individual, health system and social drivers of early hysterectomy in India and to explore the effects on women through the life course. We will conduct large-scale surveys of women ages 25–49 years in three Indian states with high prevalence of hysterectomy. Subsequently, we will conduct in-depth qualitative interviews with women who have undergone hysterectomy or sought gynaecological treatment, along with health care providers and other influences in women’s lives. We will combine these findings with evidence syntheses and consultations with women’s groups, health care providers and policymakers.

This study will contribute first-time evidence on hysterectomy and women's health in India, with a focus on understanding influences on women's health through the life course. We will gain insights into women's treatment choices and health care options when faced with gynaecological morbidity, as well as the consequences of undergoing early hysterectomy on physical and social well-being. Bringing together a wide range of evidence, we aim to provide policy-relevant insights on potential interventions to improve women’s health in India and similar settings.

## Introduction

In many countries, the most common non-obstetric surgery amongst women is hysterectomy – the surgical removal of the uterus ^1^. It is sometimes accompanied by removal of one or both fallopian tubes and ovaries (uni- or bilateral salpingo-oophorectomy). Most commonly, younger women undergo hysterectomy for non-cancerous indications such as fibroids, endometriosis and abnormal uterine bleeding, while uterine prolapse and gynaecological cancer are leading reasons for uterine removal in women older than 55 years ^1, 2^. There is no global benchmark for an appropriate population prevalence of hysterectomy. Prevalence estimates vary widely between countries as well as within countries, from 3 percent in rural China to 21 percent of adult women in the United States ^3, 4^. Evidence suggests a higher risk of hysterectomy amongst disadvantaged women, such as those with lower education and socioeconomic status ^5, 6^.

When required, hysterectomy is a critical intervention for women’s health. However, systematic reviews, mainly from high-income settings, have highlighted a range of risks associated with hysterectomy, particularly when conducted before age 45 or before menopause (early hysterectomy) ^7, 8^. Studies from Australia, the United Kingdom and the United States, for example, have found that women who undergo early hysterectomy face increased risk of diabetes, hypertension, depression, metabolic disorders and dementia ^9– 12^. Risks to women’s long-term health are higher when both ovaries are removed concurrently, which induces surgical menopause and contributes to higher risk of cardiovascular disease and all-cause mortality ^13, 14^.

Population-level data on hysterectomy in low-and middle-income countries are limited. To our knowledge, only India collects information on hysterectomy through nationally representative population surveys. In 2015–16, the first time such data were collected, the National Family Health Survey (NFHS) amongst women ages 15–49 reported that approximately 1 in 10 women have undergone hysterectomy by age 50 in India ^15^. Amongst women aged 40–49 years at the time of the survey, the median age at which hysterectomy was conducted was 37 years—approximately a decade earlier than natural menopause ^15, 16^. Further, prevalence varies widely by state, with higher prevalence in Andhra Pradesh and Telangana (up to 1 in 5 women), followed by Bihar and Gujarat ( Figure 1a) ^17^. Patterns have not changed in the subsequent survey conducted in 2019–2021 ^18^. The Longitudinal Aging Study of India (LASI), 2017–2018 amongst women >45 years reported highest prevalence in Punjab and Andhra Pradesh ( Figure 1b) ^19^.

**Figure 1a.**
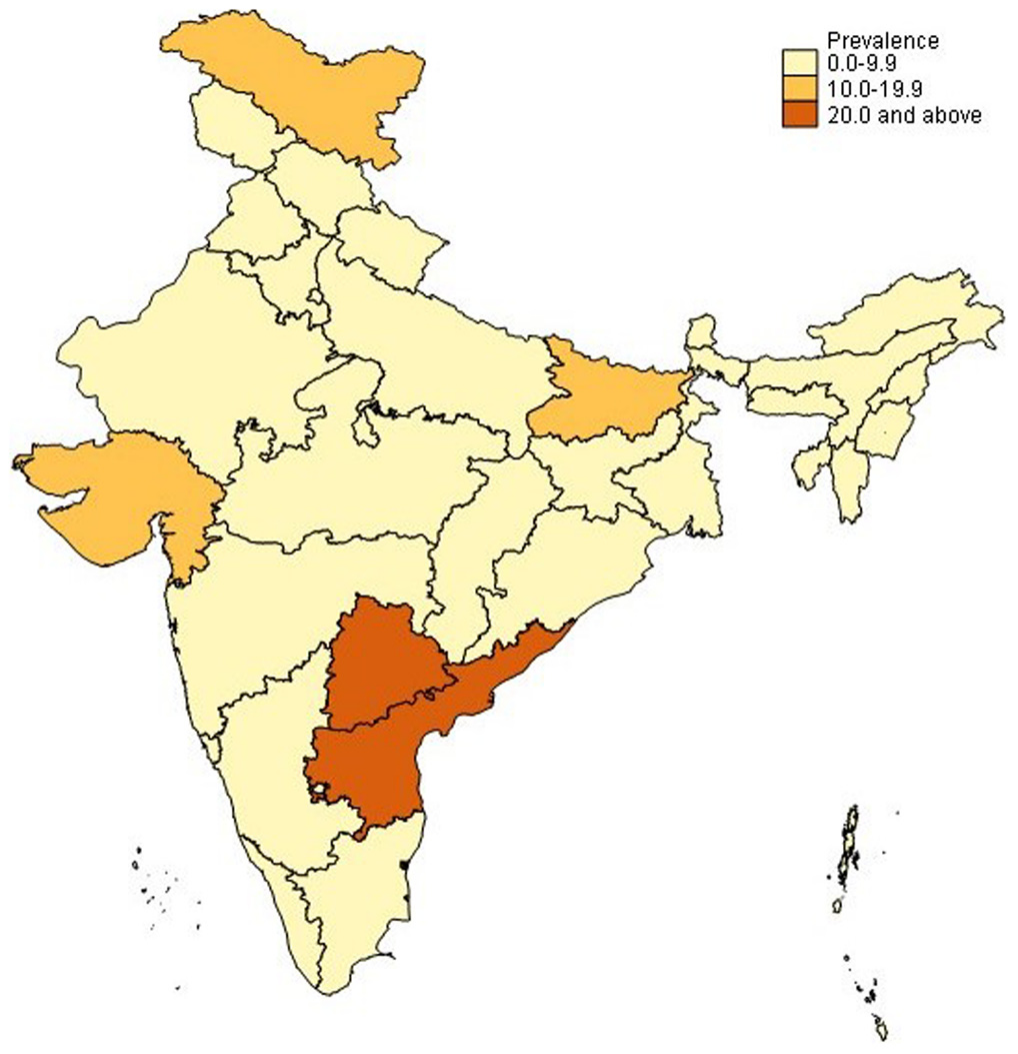
Prevalence of hysterectomy (women ages 40–49 years); NFHS-5, 2019–2021. Prevalence of hysterectomy (women ages 40–49 years); NFHS-5, 2019–2021. Source: Data from the National Family Health Survey-5, 2019–2021 ^18^. Map created by authors using Stata version 15.1 and shapefiles provided with the data.

**Figure 1b.**
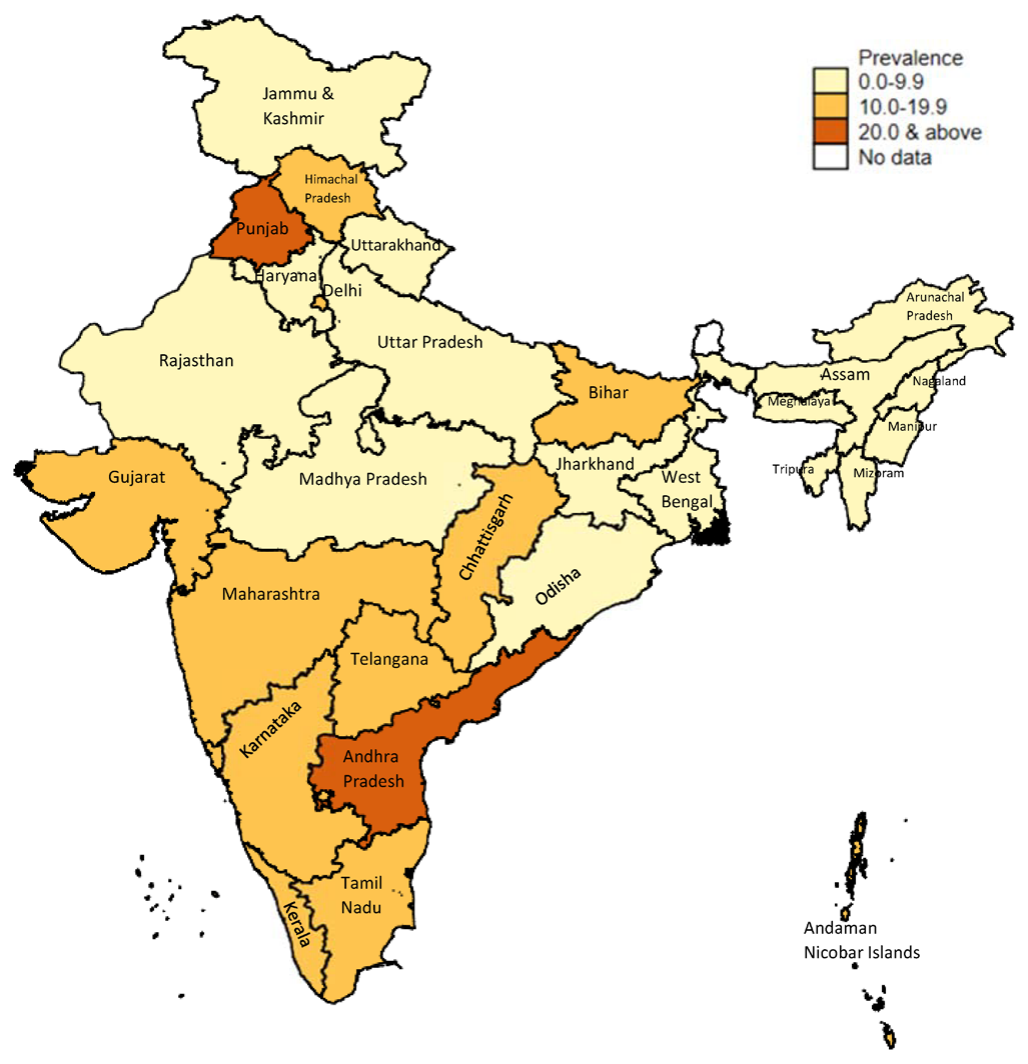
Prevalence of hysterectomy (women ages 45+); LASI-wave 1, 2017–18. Source: Data from the Longitudinal Ageing Survey of India-wave 1, 2017–18. Map created by authors using Stata version 15.1 and shapefiles provided with the data.

Hysterectomy also reflects population-level inequities in women’s access to health care: odds of undergoing the procedure were higher amongst rural women, while women with at least ten years’ schooling were less likely to undergo hysterectomy ^15^. While there are no systematic data on oophorectomy, audits from large hospitals have reported that between 38% to 59% of cases included ovarian removal, with limited information on the extent ^20, 21^. An analysis of LASI indicated an association between having had a hysterectomy and hypertension, high cholesterol, diabetes and bone/joint disease, as well as higher odds of any hospitalisation in the past year ^19^.

Low median age at hysterectomy, variation across state health systems and higher prevalence amongst disadvantaged women have critical implications for women’s health in India. In 2023, India introduced National Guidelines on Preventing Unnecessary Hysterectomy, aimed at improving the quality of gynaecological care ^22^. The guidelines include: national, state and district-level monitoring of hysterectomies, particularly amongst women <40 years; standard treatment guidelines for common gynaecological conditions such as abnormal uterine bleeding and fibroids to prevent unindicated hysterectomy; and guidance for improved outreach and primary care to address women’s gynaecological needs.

Interventions to reduce early hysterectomy must address individual and social determinants of risk, clinical practice and health systems capacity, while keeping women’s health and rights at the centre. Although evidence is emerging, there are critical gaps in knowledge to ensure that policy and programmes address women’s needs and contexts. Geographic variation in prevalence does not appear to be linked to health system performance, financing options or sociodemographic characteristics; it remains unclear what drives state-level differences. National level data in India do not allow for a life course analysis that links reproductive events, causes of hysterectomy and their consequences to women’s longer term health outcomes. Further, the design and implementation of feasible interventions requires nuanced understanding of existing treatment pathways and how they vary, particularly to inform clinical and community-based strategies.

SAHELI - Study and Action on Hysterectomy: Evidence on women’s health through the Life course in India - was designed to address these evidence gaps, aligned with policy priorities for women’s health in India, through examining: (i) individual, social and health system determinants of early hysterectomy; (ii) treatment pathways that determine women’s journeys from clinical diagnosis to hysterectomy; and (iii) the consequences of undergoing hysterectomy on women’s physical, mental, economic and social well-being across the life course. We aim to use these findings to inform feasible health system, clinical and community interventions at the primary, secondary and tertiary levels for better access to care in the Indian context.

## Methods

### Study design

**Figure 2.**
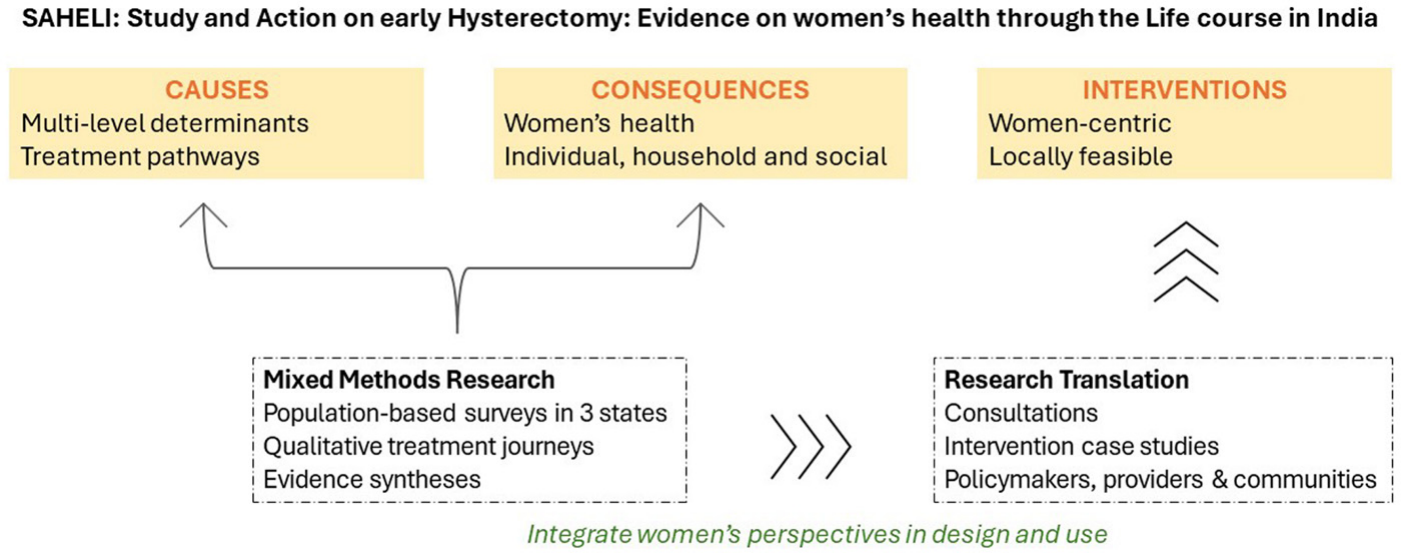
SAHELI study design. Source: Authors.

SAHELI is grounded in a feminist epidemiology approach, exploring both individual and structural sources of risk and vulnerability, with an explicit aim to integrate women’s perspectives in problem definition and knowledge production across different contexts ^23^. The study will employ a mixed-methods approach to examine individual, social, and health system factors associated with hysterectomy and women’s health. We will conduct a quantitative survey with women aged 25–49 years to examine the causes and consequences of early hysterectomy—with a focus on understanding women’s overall health, well-being and treatment-seeking patterns, followed by in-depth qualitative research with women, providers and key informants and influencers ( Figure 2). Equity-oriented health systems research methods will be used to examine national data and inform data collection ^24^, along with implementation research methods to explore the feasibility of interventions from the perspectives of communities, providers, women service users and policymakers ^25^. We will establish state-level “hubs” that include local health care providers, women’s community-based organisations and researchers to provide gain locally grounded insights and to advance research translation activities. This protocol follows the relevant sections of the STROBE reporting guidelines for observational studies.

### Setting

This five-year study (2023–2028) will be conducted in three states of India: Telangana, Bihar and Punjab. These states were selected because they report amongst the highest hysterectomy prevalence in India and represent diverse health system and sociodemographic profiles. After formative research and consultations in Year 1, the team will recruit participants and conduct quantitative surveys in Year 2 (early 2025), followed by qualitative research with women and providers in 2026-2027 and implementation research/research translation activities in the final two years. The study components are detailed below.

***1.   Formative research***

In a formative phase, we will engage women, providers and community-based organisations in stakeholder consultations through key informant interviews and group discussions in each state. The formative research will also identify local vocabulary for anatomy and gynaecological morbidity as used by women in different contexts within India, explore effective ways to capture women’s health and gynaecological issues in the quantitative survey and qualitative research and to identify new themes to include in the overall research study.

***2.   Population-based surveys***

We will recruit a population-representative sample of a minimum of 3300 women (1100 per state) from the three states (Telangana, Bihar, and Punjab). The study will recruit women from age 25 years onwards to capture reproductive life events, including menstrual health, pregnancy outcomes, caesarean section, sterilisation, gynaecological morbidity and menopause. We will enrol women at an upper age limit of 49 years, close to the age of natural menopause and the age after which use of hysterectomy decreases ^16, 26^.

We estimated the sample size based on (i) sufficient precision to estimate the prevalence of gynaecological morbidity. The District Level Health Survey conducted in 2007–08 indicated that between 19 and 22% of women in India aged 25-49 years reported a menstrual problem, with more than 11% reporting excessive bleeding ^27^ and (ii) sufficient matched pairs to examine associations between hysterectomy and common chronic conditions, e.g. hypertension, matched for age and socioeconomic status. We assumed a prevalence of 15% women with excessive menstrual bleeding, with a design effect of 2 and margin of error of 3% with 95% confidence interval.

Accordingly, where d=margin of error; p=proportion of population with a specific prevalence; and *α*=level of significance (5%), the sample size calculation is:


$$\text{N}=1.96^{2}*\frac{0.15*0.85}{0.03^{2}}*2=1088\;(\text{minimum}\;\text{sample}\;\text{size})=1100\;\text{women}\;\text{per}\;\text{state}$$

We will include all women in the age group 25-49 years, which covers any age-eligible woman per household. Considering a non-response of 15%, 163 additional households will be selected. The estimated sample will include between 80 and 150 women with hysterectomy in each state. To ensure sufficient matched pairs to examine associations between hysterectomy and common chronic conditions, e.g. hypertension, matched for age and socioeconomic status, we will recruit (oversample) an additional 500 women with hysterectomy per state.

SAHELI will adopt a multistage stratified random sampling design to select study participants. The first stage will select primary sampling units (PSUs), i.e., sub-districts. Sub-districts of each state will be stratified into high, medium and low categories, based on the prevalence of hysterectomy in women 25-49 years (mean ± SD). Proportionate numbers of PSUs from each hysterectomy stratum will be selected following explicit stratification criteria of (i) household size and (ii) percentage of scheduled caste and scheduled tribe populations as well as the implicit criterion of female literacy rate, using the probability proportionate to size (PPS) method.

In the second stage, using the PPS method, four secondary sampling units (SSUs), i.e., villages in rural areas, and wards in urban areas, will be selected from each PSU. These four SSUs will be divided in rural and urban SSUs proportional to the PSUs’ rural-urban population distribution. Additionally, SSUs with fewer than 50 households (HHs)will be excluded from the frame. In larger SSUs with more than 500 HHs, segments of 150 HHs will be created, of which two segments will be selected randomly using the PPS method. In urban areas, after the selection of wards, one census enumeration block (CEB) will be randomly selected as a tertiary sampling unit (TSU). To develop a sampling frame, a male mapper will identify all households of each SSU/TSU. A female lister will visit these households to list information on household size, the number of women aged 25–49 years and the number and age of women who have undergone hysterectomy. In the final stage, a fixed number of households from each SSU/TSU list will be selected using simple systematic random sampling. The additional sample of 500 women who have undergone hysterectomy from each state will be drawn from the comprehensive list prepared during the house listing process.

The survey will assess women’s overall health through self-reports and biomarker screening. It will adopt a life course approach that includes data on: demographic characteristics including employment; childhood exposures; reproductive histories (e.g., age at menarche, menstrual health, age at first childbirth, pregnancy outcomes and use of contraception); gynaecological morbidity during different life phases; diet and nutritional status; individual and household decision-making power; household environment; time-use; treatment pathways for gynaecological morbidity; self-reported physical and mental health status; and quality of life. Women who consent will undergo anthropometric measurements and be screened for hypertension with a blood pressure monitor and cuff and for diabetes (random blood glucose) with a glucometer. We will include an in-depth module for women who have undergone hysterectomy to assess quality of life, mental health and access to treatment for side effects. Data will be analysed to examine the prevalence of and risk factors for gynaecological morbidity, explore treatment pathways, identify risk factors associated with hysterectomy and explore the consequences of undergoing the procedure.

***3.   Treatment journeys drawing on women’s and providers’ perspectives***

We will select a sub-sample of women with hysterectomy from the survey (N=120; ~40 per state) to map their treatment journeys and understand the impact of hysterectomy on their physical and mental well-being. They will be purposively selected, with variation by public and private sector facilities and socio-economic status. We will also conduct similar interviews with women who have experienced gynaecological morbidity but did not undergo hysterectomy (N=30; ~10 per state), subject to thematic saturation. Domains of inquiry will include household and health-related decision-making, diagnosis, counselling [individual and family] and treatment offered for gynaecological morbidity, informed consent, discharge and follow-up, as well as effects and consequences on women’s physical, mental and psychosocial health and socioeconomic security. In addition, we will conduct key informant interviews with front-line health workers, women’s group leaders, members of village health committees and family members to explore social norms and influences on treatment-seeking patterns (N=30; ~10 per state).

We will conduct in-depth interviews with health care providers, inclusive of gynaecologists and surgeons in the public and private sectors, who conduct hysterectomy, to understand clinical perspectives on treatment for gynaecological morbidity (N=60; ~20 per state). We will examine provider motivations including social norms, trust and patient-provider relationships and professional accountability. Providers will be identified through facility mapping and survey data.

We will integrate quantitative and qualitative methods in an sequential, iterative approach to identify convergence, dissonance and gaps ^28, 29^. We will code narrative data using a framework such as Ritchie and Spencer’s framework method for applied policy research to identify individual, household, social and health system determinants of hysterectomy and consequences ^30^. We will also create treatment journey maps ^31^ and explore grouping these into pattern typologies across states. Lastly, we will compare women’s treatment journeys to standard treatment guidelines for gynaecological morbidity in India ^32^.

***4.   Evidence syntheses on the causes and consequences of hysterectomy in India and feasible interventions***

We will conduct three evidence syntheses related to causes and consequences of hysterectomy. First, we will synthesise evidence from facility-based medical audits of hysterectomy in India to understand the clinical indications and procedures used in the country. Second, we will conduct a scoping review of evidence on consequences of hysterectomy in low- and middle-income settings and conduct a systematic review, if sufficient literature is available. Third, we will carry out a scoping review to identify feasible interventions, such as adapted clinical guidelines, reporting and audit systems, provider engagement and community-based strategies to address social norms that have been used in India and similar settings. We will place emphasis on existing treatment guidelines, interventions and surveillance/audit systems that have reduced early hysterectomy.

***5.   Consultations, intervention mapping and Delphi-based priority-setting***

SAHELI will organise consultations with stakeholders at the national level and in Telangana, Bihar and Punjab to reflect on the implications of SAHELI’s findings for health policy and programmes. Consultations will include community-based organisations, health care providers and associations, policymakers and researchers.

We will develop intervention case studies of relevant interventions in India, which are likely to include: SEWA’s education and insurance interventions ^33, 34^; clinical governance implemented by Mahatma Gandhi Institute for Medical Sciences, Wardha; the Federation of Obstetric and Gynaecological Societies of India’s (FOGSI’s) ‘Save the Uterus’ campaign, and clinical strategies used by interviewed providers. Case studies will be developed using in-depth interviews conducted with public and private health care provider bodies, government policymakers, insurance industry and regulators, professional associations and civil society groups. We will build on longstanding experience with women’s groups to identify participatory approaches to address social determinants, particularly the normalization of hysterectomy. Findings will be synthesised collaboratively to develop evidence-based, feasible recommendations. Lastly, we will engage in co-production and prioritisation of interventions with stakeholders adapting a Delphi methodology ^24, 35^, to understand the feasibility of clinical, public health and policy interventions to address the causes and consequences of early hysterectomy in India.

## Dissemination

This research programme aims to inform clinical and practice guidelines and influence policy development and priorities for women’s health through the life course in India. Drawing on our consultations, we will develop tailored dissemination strategies for different stakeholders. At the policy level, we will aim to co-produce strategies to address early hysterectomy and improve the quality of gynaecological care and women’s health services, through policy briefs and detailed protocols for different health system levels in partnership with expert groups. For researchers, we will develop peer-reviewed publications on the causes and consequences of hysterectomy and potential intervention strategies. For women’s health advocates and women’s groups, we will organise events to initiate and sustain public discourse on access to quality gynaecological care and promoting women’s health through the life course, with engagement of women affected by early hysterectomy. Dissemination events with women’s groups, researchers and policymakers will be organised at the state level in partnership with state-level organisations. We will develop a study website, along with media engagement to share findings with the broader public. Lastly, we will explore developing mixed media tools, such as a short film, podcasts, or booklets on SAHELI’s findings and their implications for policy and interventions.

## Discussion

SAHELI aims to generate new, policy-relevant evidence on hysterectomy and women’s health through the life course in India through a multi-disciplinary programme of work that includes (i) population-level research (ii) mixed-methods health systems research with women and health care providers and (iii) and knowledge translation to inform policy priorities. The study will provide unique, first-time insight into the burden of different types of gynaecological morbidity amongst Indian women, and the nature, quality and extent of treatment options and pathways chosen. The study will also establish a women’s health cohort in India that can create a basis for future research.

The key strengths of this study are its basis in feminist principles, the use of a mixed-methods and life course approach and an intentional set of activities to inform policy priorities and interventions on women’s health in India. Conducted by a multi-disciplinary team, the study will prioritize the voices and experiences of women at every stage. We aim to co-develop evidence as well as collaboratively identify concrete actions that stem from our findings.

One limitation is the cross-sectional, rather than longitudinal, design of the quantitative research which hinders causal inference on the determinants of hysterectomy. However, the large sample size and qualitative research will provide substantive insights that can indicate likely factors at the individual and state level. Moreover, we hope that this study is the initiation of longer-term cohort studies comprising the same population. Also, SAHELI does not include primary research in low-prevalence states, which should be included in future research to further understand geographical variation.

This study aims to provide evidence from three diverse Indian states that will capture a combination of epidemiological patterns, social norms and health system factors that drive early hysterectomy in similar settings including those beyond India. Further, it will add diversity to the emerging evidence base, largely from high-income studies, on the consequences of early hysterectomy. With rapidly expanding access to surgery and shifting epidemiological profiles across the world, it is critical that evidence on women’s health examines the causes and consequences of common gynaecological procedures, across the life course and across settings.

## Ethics and consent

The study has received ethics approval from the Institute Ethics Committee, All India Institute of Medical Sciences (AIIMS), New Delhi (IEC 499/03.08.2023, RP-20/2023) on 14.8.2023, and Institute Ethic Committee, AIIMS Patna (AIIMS/Pat/lEC/2022/974) on 25.11.2022 for the overall research study. The study team has also received approval for the formative research component from the Institutional Review Board, International Institute for Population Sciences, Mumbai (IIPS/PSC-54/IRB/401/2024) on 2.1.2024. The study team will subsequently apply for ethics approval for the survey and qualitative research components prior to initiation. Ethics approval from additional partner institutions will be sought at the state level.

Ethics procedures include anonymity, voluntary participation and right to withdrawal. Written consent (signature or thumb imprint) will be obtained from study participants by trained research interviewers before any study procedures (i.e., surveys, examinations, and discussions) are initiated. Participation in the study will be completely voluntary and participants may refuse to answer any or all questions. They will have the right to withdraw from the study at any time. All information shared will remain anonymous and confidential; data will be de-identified prior to analysis. Group discussion participants will be requested to maintain the confidentiality of the discussion and identities of their fellow discussants. All collected consent forms will remain confidential; documents will be securely maintained for five years after data collection, as per standard institutional procedures.

In addition to these standard ethics procedures, the SAHELI team is developing processes to refer women involved in any stage or aspect of the study to treatment or follow-up, particularly for women who require care for gynaecological morbidity or post-hysterectomy conditions. We will develop a list of local gynaecologists, particularly those in the government sector who provide free services, provide women with information, and as feasible, create direct linkages with providers who can offer treatment options.

## Data Availability

No data are associated with this article.
