# Supplementary material for: SAHELI:  Study and Action on Hysterectomy: Evidence on women’s health through the life course in India.  Protocol for a mixed-methods study
Source: Wellcome Open Res. 2025 Mar 5;9:584. Originally published 2024 Oct 15. [Version 2] doi: 10.12688/wellcomeopenres.23084.2 (PMC11803392; doi:10.12688/wellcomeopenres.23084.2)
Supplement: Peer review history

# Reviewer report: Reviewer response for version 1

**DOI:** 10.21956/wellcomeopenres.25420.r115543

**Published:** 2024-12-21

**Authors:** Elizabeth Bertone-Johnson

**Affiliations:** 1 Health Promotion and Policy, University of Massachusetts, Amherst, MA, USA

Thank you for inviting me to review the protocol for this project. The study addresses early hysterectomy, an important topic for women’s long-term health in India. Its use of mixed methods, including quantitative surveys and participant interviews to map treatment journeys, is particularly innovative. Results from this work will further our understanding of the epidemiology of early hysterectomy and provide targets for clinical and policy-based interventions.  The protocol is very well written, comprehensive and compelling. My only suggestion is for the authors to clarify where they are in the research process. The protocol states that the 5-year study runs from 2023-2028, and it would be useful to know what phases of the work are underway and which have been completed as of 12/31/24.

Is the study design appropriate for the research question?

Yes

Is the rationale for, and objectives of, the study clearly described?

Yes

Are sufficient details of the methods provided to allow replication by others?

Yes

Are the datasets clearly presented in a useable and accessible format?

Yes

Reviewer Expertise:

Epidemiology, women's health, menopause, mental health

I confirm that I have read this submission and believe that I have an appropriate level of expertise to confirm that it is of an acceptable scientific standard.

# Response

**Published:** 2025-02-21

**Authors:** Sapna Desai

**Affiliations:** Population Council Institute, New Delhi, Delhi, India

Thank you for your encouraging review of this protocol.  As of December 2024, we  completed our inception phase, including formative research and community engagement.  We just finalised research sites, and are currently preparing for the survey to be initiated in March 2025.  We will also update the study protocol after this phase is completed.

# Reviewer report: Reviewer response for version 1

**DOI:** 10.21956/wellcomeopenres.25420.r110281

**Published:** 2024-11-26

**Authors:** Anupama Bahadur

**Affiliations:** 1 All India Institute of Medical Sciences, Rishikesh, India

A very thought provoking topic in a country like India where uterus is considered a vestigial organ once its function as a reproductive organ is complete. The three states selected are apt for the study. Young women are subjected to hysterectomy at the slightest of indication including discharge per vaginum. At times even without complete evaluation a woman's uterus is removed and we have a histopathology report stating malignancy of the genital tract. It is sad to see how these cases are mismanaged. Therefore this study will bring forth important key points which can help in formulating guidelines at central level. Morbidity and mortality if any must be documented to generate evidence from the data of more than 33,000 women. Look forward to the results of this study.

Is the study design appropriate for the research question?

Yes

Is the rationale for, and objectives of, the study clearly described?

Yes

Are sufficient details of the methods provided to allow replication by others?

Yes

Are the datasets clearly presented in a useable and accessible format?

Yes

Reviewer Expertise:

I am working as Professor and Unit Head at AIIMS Rishikesh and find this study of utmost importance in our country where the uterus is removed at the slightest indication but the same is not true with the male counterpart. We need a law counteract the menace of hysterectomy in our country where this procedure is financially driven. The function of the uterus is childbearing and once this function is over it doesn't take much to remove it by applying four clamps and disconnecting it from the pedicles. I am sure that if our Institute is also included as a part of this study we can contribute to meaningful data from the state of Uttarakhand.

I confirm that I have read this submission and believe that I have an appropriate level of expertise to confirm that it is of an acceptable scientific standard.

# Response

**Published:** 2025-02-21

**Authors:** Sapna Desai

**Affiliations:** Population Council Institute, New Delhi, Delhi, India

Thank you for your encouraging responses.  We will ensure to share our findings with you and the gynaecologist community early on, to ensure the study can support development of feasible interventions to address early hysterectomy that is not medically indicated.
